# Transcaval Access for Impella Placement: A Viable Alternative in Appropriate Patients

**DOI:** 10.1016/j.jscai.2025.103855

**Published:** 2025-07-31

**Authors:** Grant W. Reed, Ankur Kalra, Fawzi Zghyer

**Affiliations:** a Department of Cardiovascular Medicine, Cleveland Clinic, Cleveland, Ohio; b Division of Cardiology, Department of Medicine, SUNY Upstate Medical University, Syracuse, New York

**Keywords:** Impella, mechanical circulatory support, transcaval access

The landscape of mechanical circulatory support (MCS) for treatment of acute myocardial infarction complicated by cardiogenic shock has incrementally evolved with the advent of percutaneous ventricular assist devices. These devices offer crucial hemodynamic support when noninvasive therapies fall short. Yet, one of the most important limitations to broader utilization of MCS devices including Impella CP (Abiomed) remains frequent vascular access complications including bleeding and acute limb ischemia. Patients with severe peripheral arterial disease (PAD), small-caliber or tortuous iliofemoral arterial vasculature, or previous peripheral interventions present a major access challenge. While axillary artery access offers an alternative, it typically requires surgical exposure for placement and removal and may not be readily available in an emergent setting. Antegrade sheath placement into the superficial femoral artery for limb perfusion during femoral Impella use is sometimes used, but it is often impractical and can be challenging in the acute setting. Limb ischemia remains a significant concern in patients with PAD, even when best practices are followed.^1^ In the DanGer Shock trial, limb ischemia was reported in 5.6% of patients treated with Impella, compared with 1.1% in those managed with standard of care, reinforcing the importance of considering alternative access strategies in appropriately selected cases.^2^

In this context, transcaval access (TCA) for Impella insertion has attracted interest. Most widely reported as an alternative for transcatheter aortic valve replacement (TAVR) in patients with no suitable femoral arterial access, the approach involves crossing from the inferior vena cava to the adjacent abdominal aorta to create an arterial access route.^3^ In experienced hands, this technique is effective, with similar bleeding complication rates to other alternative access routes. Accordingly, the 2022 and 2023 Society for Cardiovascular Angiography & Interventions (SCAI) consensus documents acknowledge the growing role of alternative access strategies.^4^ Similarly, the 2025 SCAI Expert Consensus on Alternative Access TAVR recognizes the potential role of TCA and highlights the importance of institutional expertise in access planning and complication management.^5^

This study by Mohammed et al^6^ is therefore timely and thought-provoking. It presents the largest experience to date of Impella 5.0 delivery via TCA in cardiogenic shock, encompassing 72 patients over an 8-year period. The authors’ report 100% technical success, low access-related complication rates, and a 44% in-hospital survival—outcomes that are notable given the acuity of illness of this population. Importantly, almost all cases were performed without preprocedural computed tomography imaging, using only fluoroscopic landmarks to guide access at L3-L5, and yet the procedural success and safety were preserved.

The study stands out for its practical procedural detail, real-world relevance, and thoughtful discussion of the tradeoffs inherent to alternative access strategies for Impella placement. The inclusion of both ischemic and nonischemic cardiomyopathy patients, and the clear articulation of indications for TCA (small femoral arteries and previous access complications) enhance the applicability of the findings. Unlike standard femoral arterial access, which carries a risk of limb ischemia and bleeding requiring transfusion or intervention, TCA complications were uncommon and largely manageable with nitinol occluders or covered stents. Only 1 death (1.4%) was attributed to access-related bleeding.

Despite its strengths, certain limitations should be acknowledged. This is a single-center study, and as the authors mention, the generalizability outside of expert centers is uncertain. Transcaval puncture, wire electrification, and aortic closure require technical proficiency. Similar to alternative access TAVR, proctoring is essential for safe adoption. While not common in this study, computed tomography imaging to assure there is a safe inferior vena cava–aorta puncture zone without significant aortic calcification may further improve procedural safety. It is also important to note that the Impella 5.0 device is no longer commercially available in the United States, although this does not diminish the feasibility of the technique the authors demonstrate.

While the current study focuses on patients with poor femoral access, given the favorable outcomes, a provocative question emerges: should TCA be considered more broadly for Impella insertion, even in patients with adequate femoral access? Several important considerations must be weighed when considering TCA for Impella placement (Table 1). In theory, TCA has several advantages. Unlike femoral arterial access, TCA avoids arterial limb ischemia entirely. The need for antegrade sheath placement to preserve limb perfusion is eliminated. As acute limb ischemia is the Achilles heel of Impella CP placement, this could represent a significant step forward in reducing complication rates. It is feasible that vascular complication rates would be lower in patients without significant PAD, as strategies to deal with aortic complications (eg, Coda balloon or endograft delivery) depend on adequate contralateral femoral access.

**Table 1:** Important considerations in transcaval access for Impella insertion.

Advantages (Pro)	Disadvantages (Con)
• Allows for percutaneous femoral Impella placement in patients with advanced PAD • Eliminates risk of acute limb ischemia • Reduced risk of femoral bleeding • Allows for larger MCS devices via a femoral approach (Impella 5.5) • Potentially applicable to other large-bore access procedures (TAVR, VA-ECMO, etc) • Possible alternative to axillary Impella 5.5 placement in appropriate patients	• Requires expertise in caval-aortic puncture, electrocautery, and closure devices • More time consuming than traditional femoral access for Impella CP • Potential for venous access complications • Requires immobilization, bed rest (in contrast to axillary access) • Potentially unable to “bail-out” aortic complications if significant PAD • Data limited to single-center experience • Proctoring should be strongly considered prior to starting a program

MCS, mechanical circulatory support; PAD, peripheral arterial disease; TAVR, transcatheter aortic valve replacement; VA-ECMO, venoarterial extracorporeal membrane oxygenation.

Extrapolating the findings of this study, TCA may be relevant to the delivery of other MCS devices, including venoarterial extracorporeal membrane oxygenation and newer-generation MCS platforms in development. It is feasible that TCA may allow for femoral implantation of the larger Impella 5.5 device, which is usually surgically implanted via axillary access. Prospective and randomized studies are needed to clarify the relative safety profiles of TCA versus traditional access routes for MCS delivery. Transcaval TAVR studies, for instance, report major vascular complications in 7% to 13% of cases, although often in preplanned, computed tomography guided contexts.^7^^,^^8^ A comparative summary of complication profiles could inform clinical protocols and access strategy recommendations.^5^

While enthusiasm should be tempered based on findings from a single-center experience, this study demonstrates that when performed at a center of excellence, TCA for Impella delivery is not only feasible but may also offer a safer alternative in patients with challenging femoral arterial anatomy. The authors should be congratulated on this important study, which has implications that extend beyond Impella 5.0 placement, particularly given the growing interest in delivering larger MCS devices via percutaneous means. That said, widespread adoption should proceed cautiously and only after establishing training infrastructure, case selection guidance, and consideration of a multicenter registry or prospective trials. As the field of interventional cardiology continues to refine optimal practices for the delivery of MCS devices in cardiogenic shock, novel access strategies including TCA may help shift the paradigm, not just for those without options, but potentially for many more.
